# Vocal emotion recognition: A comparison of singers and instrumentalists, amateurs and professionals

**DOI:** 10.1016/j.isci.2025.114089

**Published:** 2025-11-17

**Authors:** Christine Nussbaum, Jessica Dethloff, Annett Schirmer, Stefan R. Schweinberger

**Affiliations:** 1 Department for General Psychology and Cognitive Neuroscience, Friedrich Schiller University Jena, Jena, Germany; 2 Voice Research Unit, Friedrich Schiller University, Jena, Germany; 3 Institute of Psychology, University of Innsbruck, Innsbruck, Austria; 4 Swiss Center for Affective Sciences, University of Geneva, Geneva, Switzerland

**Keywords:** Sensory neuroscience, Cognitive neuroscience

## Abstract

Musicians outperform non-musicians in vocal emotion recognition (VER), presumably due to differences in auditory sensitivity. However, the current literature is inconclusive regarding differential effects of specific types of musical activity. Therefore, we compared emotion recognition performance of singers (*N* = 45) vs. instrumentalists (*N* = 43) and professional musicians (*N* = 40) vs. amateurs (*N* = 88) vs. non-musicians (*N* = 38), based on short vocal utterances expressing happiness, pleasure, fear, or sadness. Using both frequentist and Bayesian inference, we found the predicted null effects for singers vs. instrumentalists, and professionals vs. amateurs. Evidence for an advantage in amateurs vs. non-musicians was inconclusive. Across groups, we replicated the consistent link between VER and auditory sensitivity. Overall, the current work aligns with the perspective that musicians’ advantage in recognizing vocal emotions is rooted in auditory sensitivity, rather than specific types of musical activities or formal training.

## Highlights

• We investigated vocal emotion recognition in subgroups of musicians
• Vocal emotion recognition did not differ between singers vs. instrumentalists
• Vocal emotion recognition did not differ between amateurs vs. professionals
• We replicated the link between auditory sensitivity and vocal emotion recognition

## Graphical abstract

**Figure:**
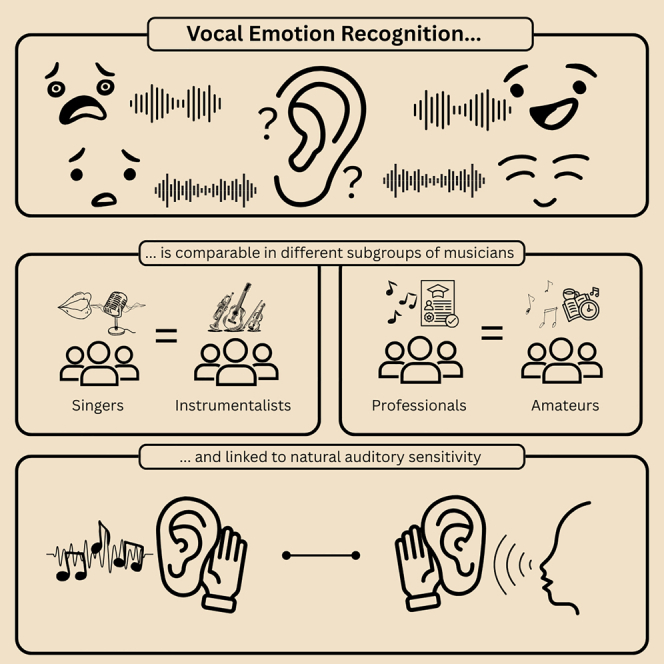

## Introduction

### Associations between musicality and vocal emotion recognition

The human voice is a prime carrier of emotional information. Therefore, adequate perception of vocal emotions is important for everyday social interaction.^1^^,^^2^ On average, humans can infer emotions from voices well above chance,^3^^,^^4^^,^^5^ but this capacity is subject to great individual variability and seems to be linked to differences in *musicality*.^6^ It has been shown repeatedly that musicians outperform non-musician in vocal emotion recognition (VER), although the overall effect size can be considered small to moderate.^7^^,^^8^^,^^9^ Several works sought to unravel the potential mechanisms underlying this advantage. Based on the observation that emotions expressed by voices and by music share a similar acoustic code,^3^ early theoretical frameworks like the OPERA hypothesis^10^ proposed a causal effect of musical training on voice perception skills, if specific conditions are met: an overlap in the neural circuits, precision in auditory-motor demands, as well as involvement of emotion, repetition, and attention in the musical activity. However, for VER, the OPERA hypothesis has not stood up to rigorous empirical examination.^9^^,^^11^ Instead, evidence collectively points to the role of *auditory sensitivity*, which does not seem to be causally linked to formal musical training. Compared to non-musicians, musicians have on average more fine-grained basic auditory skills, such as pitch and rhythm perception, musical memory, or signal-in-noise discrimination,^12^^,^^13^ which aids VER. Importantly, this association between auditory perception and VER was observed even in the absence of any formal musical training.^14^^,^^15^^,^^16^^,^^17^^,^^18^ Further, Correia et al.^15^ found that the link between musical training and VER was fully mediated by auditory perception skills. The presumably strongest evidence is provided by a recent randomized-controlled study in school children, which found no causal effects of musical training on VER performance.^19^ Thus, there is consensus in the literature that the observed performance difference of musicians and non-musicians is due to variations in natural auditory sensitivity rather than the result of formal musical education.^9^

In a previous study, we investigated how musicians’ auditory skills are linked to VER in more detail, by focusing on different auditory cues that transport emotional meaning.^14^ We employed parameter-specific voice morphing to create vocal stimuli that expressed emotion only through fundamental frequency contour (F0), timbre, or both. F0 is linked to dynamic pitch variation (also referred to as voice melody), whereas timbre is linked to perceived voice quality (i.e., whether it sounds harsh or gentle). Professional musicians outperformed a group of non-musicians when emotions were expressed by F0 and both cues, but not timbre alone. Thus, musicians seem to be specifically proficient at exploiting melodic patterns to infer vocal emotions.

While the available literature paints a fairly consistent picture regarding the link between musicality and VER, a key limitation inherent in most studies targeting group differences is that they treat musicians as one uniform group, which is, in fact, highly heterogeneous. On the one hand, there are quantitative differences regarding levels of expertise. On the other hand, there are qualitative differences between musicians linked to a variety of styles, genres, and forms of expression, within the scope of the Western music system and beyond. A particularly interesting distinction in the context of vocal emotions is the one between singers and instrumentalists. Singing is arguably the form of musical expression that is most closely related to vocal emotions.^20^^,^^21^ Another interesting debate revolves around differences between professional musicians and amateurs.^22^ Therefore, the present study targeted these subgroups to explore their capacity for VER. In what follows, we review current insights and outstanding research gaps, cumulating in the rationale for the present study.

### Singers versus instrumentalists

Singing and playing an instrument are both fundamental forms of musical expression in humans, but they require very different motor skills and, typically, different amounts of formal musical training.^23^^,^^24^ Crucially, singers use their voice for musical expression. This is reflected in vocal performance differences, as for example, singers outperform instrumentalists in voice imitation tasks.^25^^,^^26^ Further, neuroscientific research revealed substantial overlap between the neural circuits involved in the expression and perception of vocal information.^27^ But how does this relate to the sensitivity in the perception of vocal cues? The abovementioned OPERA hypothesis^10^ would predict that singers’ high degree of auditory-motor precision and neural overlap would lead to benefits in perception. However, this is not consistently supported by empirical findings.^28^ In fact, Martins et al.^29^ found no differences in electrophysiological responses to emotional voices between singers and instrumentalists, suggesting similar profiles of auditory processing. Apart from this study, relevant evidence regarding VER is sparse and inconclusive. Several studies observed correlations between VER and singing abilities, either self-rated or objectively measured,^14^^,^^15^^,^^30^ but all samples comprised both singers and instrumentalists. Intriguingly, a music-intervention study reported that singing may even interfere with vocal emotional processing, while instrument lessons had a positive effect.^31^ However, the validity of this finding is limited by an extensive drop-out of participants and a small sample size.^9^ Overall, the few data that are available do not provide clearcut, let alone causal evidence for a specific benefit in VER by singing over playing an instrument. We therefore pursued the null hypothesis of there being no such differences. In view of the limitations of previous studies, we recruited a well-powered sample of instrumentalists and singers.

### Amateurs versus professional musicians

Most musicians start with their formal training in childhood, but when they enter adulthood, they pick different paths: some convert their musical activity into a profession, whereas others pursue another career and keep music as a hobby. Interestingly, these groups seem to display several differences with regard to neurocognitive functioning. While amateurs, unsurprisingly, score lower on musical abilities, they show better cognitive abilities in terms of abstract reasoning than professional musicians.^22^ Amateurs may gain more positive outcomes from their musical activity. Perhaps because it takes up less time in their lives, it is enriching, while at the same time minimizing negative consequences related to, for example, exposure to high amplitude sounds and performance pressure. This also seems to be reflected in general health, which was found to be better in amateurs than professionals.^32^^,^^33^^,^^34^^,^^35^^,^^36^ On a different note, one recent study reported that professionals when compared with amateurs were more likely to experience a state of flow during their musical activity, which is usually considered very enjoyable.^37^ However, to the best of our knowledge, there have been no comparisons between amateurs and professionals with regard to VER. This gap is addressed with the present study.

### Rationale of the present study

This study focuses on the comparison between singers and instrumentalists as well as professionals and amateurs and thus zooms into possible similarities or differences between specific subgroups while using an almost identical protocol as Nussbaum et al., 2024^14^ We report our results in three parts. For part 1, we recruited an original sample of amateur instrumentalists and singers and compared their VER, their musical perception performance and self-rated musicality. In part 2, we focused on the correlations between these measures, in order to replicate the link between auditory sensitivity and VER reported by previous studies. Finally, because all our newly recruited singers and instrumentalists were amateurs, the present study offered the opportunity to compare findings with our previously recruited groups of professional musicians and non-musicians,^14^ which we report in part 3.

As mentioned earlier, we predicted that singers and instrumentalists as well as professionals and amateurs perform equally well in our VER task, both for emotions expressed by all available vocal cues, as well as emotions expressed by either F0 or timbre cues. We derived these predictions from previous research suggesting that the neural processing of vocal emotions is comparable in singers and instrumentalists.^29^ Moreover, we relied on behavioral evidence that the link between musicality and VER is not related to formal training, but rather to natural differences in auditory sensitivity.^15^^,^^19^ This study and its hypotheses have been preregistered (https://doi.org/10.17605/OSF.IO/76PV5).

## Results

### Part 1: Comparison of non-professional singers and instrumentalists

#### Hypotheses

Regarding the comparison between singers and instrumentalists, we formulated the following hypotheses:

**H1:** We expect *no* difference between singers and instrumentalists in overall VER performance.

**H2:** We expect *no* difference between singers and instrumentalists in VER performance based on timbre and F0 cues only.

#### Demography, musicality, and personality of participants

First, we checked for important demographic and psychological variables that the two groups were comparable. Singers and instrumentalists did not differ significantly in the socioeconomic status assessed via educational level, *X*^2^ (2, *N* = 88) = 1.06, *p* = 0.588; highest academic degree, *X*^2^ (7, *N* = 88) = 9.06, *p* = 0.249, and household income, *X*^2^ (4, *N* = 88) = 5.23, *p* = 0.264 (for more details, see Table S17). Further, the groups did not differ in age or positive and negative affect (assessed with the PANAS) and were comparable regarding Big Five personality traits and autistic traits. In the Gold-MSI, singers and instrumentalists scored comparatively on the general musicality score, but there were differences on two subfactors: instrumentalists scored higher on the subfactor Formal Education, while singers scored higher on Singing. In the PROMS, both groups performed comparably in all four subtests. Participant characteristics assessed via self-report and music performance in the PROMS are summarized in Table 1.

**Table 1:** Characteristics of participants—Demography, personality, and musicality

	Singers	Instrumentalists	*t*	df^a^	*p*	Cohen’s *d*	
	*M* (SD)	*M* (SD)					
Age	27.02 (8.2)	28.51 (10.6)	−0.73	78.93	0.465	−0.17 [−0.61, 0.28]	
*PANAS*							
Positive affect	3.00 (0.68)	3.11 (0.57)	−0.77	84.78	0.446	−0.17 [−0.59, 0.26]	
Negative affect	1.53 (0.47)	1.40 (0.35)	1.49	80.61	0.141	0.33 [−0.11, 0.77]	
*Big Five*							
Openness	4.04 (0.55)	3.99 (0.51)	0.46	85.96	0.647	0.10 [−0.32, 0.52]	
Conscientiousness	3.47 (0.69)	3.76 (0.70)	−1.91	85.62	0.060	−0.41 [−0.84, 0.02]	
Extraversion	3.21 (0.70)	3.00 (0.73)	1.44	85.30	0.155	0.31 [−0.12, 0.74]	
Agreeableness	3.81 (0.57)	4.01 (0.60)	−1.61	85.29	0.112	−0.35 [−0.77, 0.08]	
Neuroticism	2.74 (0.77)	2.61 (0.78)	0.80	85.75	0.426	0.17 [−0.25, 0.60]	
*AQ*							
Total	18.20 (6.15)	19.28 (8.55)	−0.68	76.04	0.500	−0.16 [−0.60, 0.30]	
Attention to detail	5.40 (2.33)	5.63 (2.53)	−0.44	84.64	0.662	−0.10 [−0.52, 0.33]	
Social	12.80 (5.37)	13.65 (7.53)	−0.61	75.65	0.545	−0.14 [−0.59, 0.31]	
Social skills	2.40 (1.94)	3.09 (2.95)	−1.30	72.01	0.200	−0.31 [−0.77, 0.16]	
Communication	2.53 (1.94)	2.44 (2.31)	0.20	82.02	0.842	0.04 [−0.39, 0.48]	
Imagination	2.51 (1.75)	2.81 (1.88)	−0.78	84.86	0.437	−0.17 [−0.60, 0.26]	
Attention switching	5.36 (1.91)	5.30 (2.23)	0.12	82.69	0.905	0.03 [−0.40, 0.46]	
*Gold-MSI*							
General ME	4.78 (0.85)	4.75 (0.80)	0.17	85.99	0.866	0.04 [−0.39, 0.46]	
Active engagement	3.83 (0.82)	4.21 (1.13)	−1.79	76.79	0.078	−0.41 [−0.86, 0.05]	
Formal education	**4.39 (1.14)**	**4.95 (0.62)**	−2.85	68.31	0.006	−0.69 [−1.18, −0.20]	∗∗
Emotion	5.50 (0.81)	5.60 (0.76)	−0.60	85.99	0.549	−0.13 [−0.55, 0.29]	
Singing	**4.98 (0.97)**	**4.19 (1.27)**	3.25	78.56	0.002	0.73 [0.27, 1.19]	∗∗
Perception	5.73 (0.82)	5.77 (1.03)	−0.22	80.11	0.825	−0.05 [−0.49, 0.39]	
*PROMS*							
Pitch	0.23 (0.08)	0.24 (0.06)	−0.30	82.92	0.766	−0.07 [−0.50, 0.37]	
Melody	0.17 (0.10)	0.14 (0.10)	1.29	85.08	0.199	0.28 [−0.15, 0.71]	
Timbre	0.29 (0.08)	0.3 (0.09)	−0.59	86.00	0.556	−0.13 [−0.55, 0.30]	
Rhythm	0.31 (0.09)	0.32 (0.09)	−0.56	85.13	0.577	−0.12 [−0.55, 0.30]	

Descriptive values show mean ratings for the PANAS,^38^ the Big-Five Domains,^39^ and the Gold-MSI.^40^ AQ score were calculated based on Hoekstra et al.^41^ and Baron-Cohen et al.^42^ Bold entries mark significant differences (*p* <. 05)

a Note that original degrees of freedom were 86 but were corrected due to unequal variance.

#### Emotion classification performance

The mean proportion of correct responses was submitted to an analysis of variance (ANOVA) with Emotion (Happiness, Pleasure, Fear, and Sadness) and Morph Type (Full, F0, and Timbre) as repeated measures factors and Group (singers and instrumentalists) as a between subject factor (see Table 2).

**Table 2:** Results of the 4 × 3 × 2 mixed-effects ANOVA on the mean proportion of correct responses

	df1	df2	*F*	*p*	Ω_p_^2^ [95% CI]	*ε*_HF_
Group	1	86	0.38	0.542	0.00 [0.00, 0.01]	–
Emotion	3	258	72.43	<0.001	0.45 [0.36, 0.52]	–
Morph Type	2	172	768.93	<0.001	0.90 [0.87, 0.92]	0.741
Group × Emotion	3	258	2.14	0.095	0.01 [0.00, 0.04]	–
Group × Morph Type	2	172	0.36	0.635	0.00 [0.00, 0.01]	–
Emotion × Morph Type	6	516	22.78	<0.001	0.20 [0.14 0.25]	0.827
Group × Emotion × Morph Type	6	516	1.33	0.249	0.00 [0.00 0.01]	–

ε_HF_, Huynh-Feldt (HF) epsilon correction factor in case of violation of the sphericity assumption.

**Figure 1:**
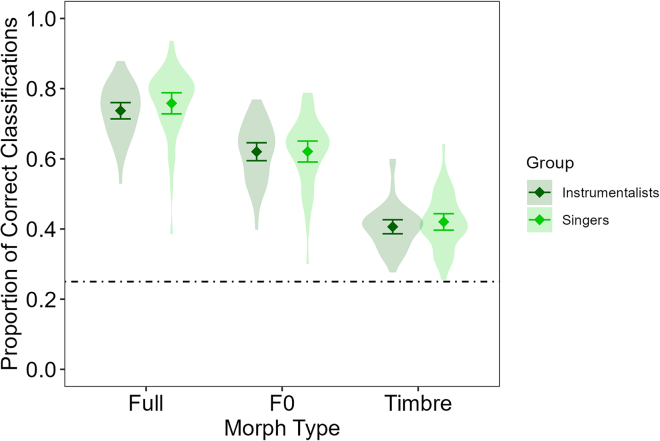
Mean proportion of correct responses per Morph Type separately for singers and instrumentalists Whiskers represent 95% confidence intervals. Violin plots represent variation of individual participants. The dotted line represents guessing rate at 0.25.

The results revealed main effects of Emotion and Morph Type, which were qualified by an interaction. Crucially, however, we found no main effects or interactions involving Group (see Figure 1), which was also confirmed by a Bayesian ANOVA (see Table S1). Planned Bayesian analysis revealed moderate evidence for the null effect of group for overall performance (*p* = 0.542, BF_10_ = 0.265), as well as for Full (*p* = 0.392, BF_10_ = 0.310), F0 (*p* = 0.935, BF_10_ = 0.226), and Timbre morphs (*p* = 0.555, BF_10_ = 0.262) separately. Thus, we found evidence consistent with our hypotheses H1 and H2.

**Figure 2:**
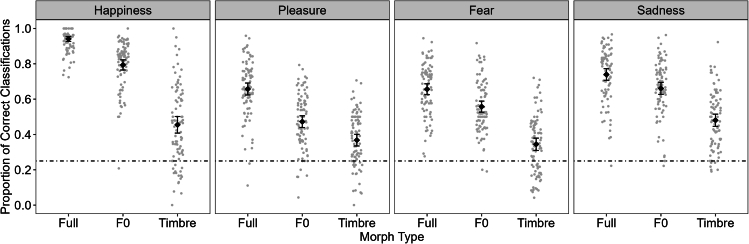
Mean proportion of correct responses per Emotion and Morph Type Whiskers represent 95% confidence intervals. Gray dots represent individual participants’ data. The dotted line represents guessing rate at 0.25.

Follow-up analysis of the Morph Type effect revealed that performance was best in the Full condition (*M* = 0.75 ± 0.01 SEM), followed by the F0 (*M* = 0.62 ± 0.01) and then the Timbre condition (*M* = 0.41 ± 0.01); Full vs. F0: |*t*(87)| = 23.28, *p* < 0.001, *d* = 2.50 [2.07, 2.92], F0 vs. Timbre: |*t*(87)| = 21.44, *p* < 0.001, *d* = 2.30 [1.90, 2.70], Full vs. Timbre: |*t*(87)| = 33.94, *p* < 0.001, *d* = 3.64 [3.06, 4.21]). This Morph Type main effect was also found for all emotions separately (all *F*s(2, 174) > 102.44, *p* < 0.001), although it differed slightly between emotions, as suggested by the interaction (see Figure 2, for all post hoc tests, refer to the full analysis on OSF: https://osf.io/ascqx/). Performance difference between F0 and Timbre was largest for Happiness (*M*_F0-Timbre_ = 0.34 ± 0.02 SEM), followed by Fear (*M*_F0-Timbre_ = 0.21 ± 0.02), Sadness (*M*_F0-Timbre_ = 0.18 ± 0.02), and Pleasure (*M*_F0-Timbre_ = 0.10 ± 0.02; all pairwise comparisons |*t*s(77)| ≥ 2.57, *p*s ≤ 0.012, *d*s ≥ 0.28 [0.06 0.49], except for Fear vs. Sadness (|*t*(87)| = 1.13, *p* = 0.261). These effects of Morph Type and Emotion, therefore, present a full replication of the patterns reported in Nussbaum et al.^14^ For confusion matrices and supplemental analyses, please refer to Figures S1–S3.

### Part 2: Correlational analyses

#### Hypotheses

In part 2, we focused on the correlations between auditory sensitivity and VER. The aim here was to see if the patterns found in Nussbaum et al.^14^ would replicate. Therefore, we formulated the following predictions.

##### Correlations with the PROMS

**H3a:** Averaged VER performance is correlated with averaged music perception performance.

**H3b:** Full-VER and F0-VER are correlated with melody perception in music.

##### Correlations with the GOLD-MSI

**H3c:** Averaged-VER and Full-VER are correlated with the general musical sophistication index (General-ME), measured by the Gold-MSI.

**H3d:** Averaged-VER and Full-VER are correlated with the perception subscale of the Gold-MSI.

**H3e:** Averaged-VER and Full-VER are correlated with self-rated singing abilities subscale of the Gold-MSI.

#### Correlations

Replicating our previous findings, we obtained a strong correlation between VER and music perception performance, as measured with the PROMS (Tables 3, S3, and S4). Further, we replicated the specific link between VER and the melody subtest. Although we did not have a specific hypothesis, we again found a link with Rhythm perception. There were no links between performance in the timbre morph condition and the timbre subtest. Overall, this pattern of correlations almost completely replicates the pattern observed in a previous sample.^14^ Thus, the link between auditory sensitivity and VER seems to be highly comparable across professional musicians, non-musicians, and amateurs.

**Table 3:** Spearman correlations between the PROMS and VER performance

	PROMS_Avg_	Pitch	Melody	Timbre	Rhythm
VER_Avg_	**0.39 (0.002)**	0.17 (0.142)	**0.29 (0.017)**	0.23 (0.050)	**0.38 (0.002)**
Full-Morphs	**0.34 (0.005)**	0.14 (0.219)	**0.27 (0.022)**	0.23 (0.050)	**0.31 (0.009)**
F0-Morphs	**0.39 (0.002)**	0.16 (0.186)	**0.34 (0.005)**	**0.24 (0.044)**	**0.32 (0.008)**
Timbre-Morphs	0.22 (0.054)	0.12 (0.305)	0.08 (0.473)	0.10 (0.352)	**0.25 (0.039)**

VER, vocal emotion recognition performance. *p* values were adjusted for multiple comparisons using the Benjamini-Hochberg correction.^43^ VER_Avg_: VER performance averaged across all trials, Full-Morphs: VER in the Full Morph condition only, F0-Morphs: VER in the F0 Morph condition only, Timbre-Morphs: VER in the Timbre Morph condition only, PROMS_Avg_: music perception performance averaged across all four subtests of the PROMS (Pitch, Melody, Timbre, and Rhythm). Bold entries mark significant correlations (*p* < .05).

In contrast, we found no links between VER performance and self-rated musicality, as measured by the Gold-MSI (all correlations ≤ 0.21, *p*s ≥ 0.051, both with and without correction for formal musical education, details on Tables S5 and S6 ). Thus, for amateurs, we could not replicate the link with self-rated musical sophistication, perception, and singing abilities, which we observed in our previous sample of professional musicians and non-musicians. Therefore, we found evidence for our hypotheses H3a and H3b, but not for hypotheses H3c–H3e.

### Part 3: Comparison of professionals, amateurs, and non-musicians

In the third part, we compared amateur musicians to both professional musicians and non-musicians. Mostly, we predicted that amateurs and professional musicians would be comparable regarding VER. However, as previous evidence reviewed above showed that amateurs can differ from professionals in cognitive abilities which could be linked to emotional sensitivity, we also considered the option that amateurs could be more proficient at making emotional inferences than professionals, reflected in H5 below. Compared to our group of non-musicians (H4), we assumed that amateurs would outperform them when emotions were expressed via full emotion cues and F0 cues only, but not timbre, because this is exactly the pattern we observed for the comparison of professional musicians and non-musicians in Nussbaum et al.^14^

#### Hypotheses

**H4:** Amateur musicians outperform non-musicians in VER, in the Full and in the F0 condition.

**H5:** Amateurs perform equal or better to professional musicians in the Full and the F0 condition.

#### Demography, musicality, and personality of participants

Again, we first confirmed that the groups were comparable on important individual variables. Professionals, amateurs, and non-musicians did not differ in the socioeconomic status assessed via educational level, χ^2^(6, *N* = 166) = 11.11, *p* = 0.085, and highest academic degree, χ^2^(16, *N* = 166) = 24.04, *p* = 0.089. However, there were differences regarding household income, χ^2^(8, *N* = 166) = 20.19, *p* = 0.010, Cramer’s *V* = 0.25, with amateurs reporting higher household income than professionals and non-musicians.

Participant characteristics are summarized in Table 4. For a full report of statistical details, please refer to Tables S7–S15 . The groups were comparable in age as well as in positive and negative affect (assessed with the PANAS). For the Big Five, analyses of variance revealed group differences for extraversion, with slightly higher levels in professionals than in amateurs. Regarding autistic traits, the three groups did not differ in their overall score, but there were differences on several subscales. In the Gold-MSI, professional musicians scored significantly higher than amateurs on all subscales, which in turn scored higher than non-musicians. This is a pattern (professionals > amateurs > non-musicians) one would expect for self-rated musicality. In the PROMS, professionals outperformed amateurs in the Pitch and Melody subtest, whereas there were no differences in the Timbre and Rhythm subtests. Amateurs performed better than non-musicians in the Pitch, Melody, and Rhythm subtest but not in the Timbre subtest. Thus, a clear pattern of professionals > amateurs > non-musicians was only found for melody and pitch.

**Table 4:** Characteristics of participants—demography, personality, and musicality

	Professionals		Amateurs		Non-musicians
	*M* (SD)		*M* (SD)		*M* (SD)
*PANAS*					
Positive affect	3.32 (0.65)		3.05 (0.63)		3.10 (0.67)
Negative affect	1.69 (0.48)		1.47 (0.42)		1.49 (0.69)
*Big Five*					
Openness	4.12 (0.50)		4.02 (0.53)		3.81 (0.80)
Conscientiousness	3.49 (0.71)		3.61 (0.70)		3.76 (0.72)
Extraversion	**3.48 (0.66)**	>	**3.11 (0.72)**		3.38 (0.79)
Agreeableness	3.92 (0.57)		3.91 (0.59)		3.75 (0.66)
Neuroticism	2.95 (0.65)		2.69 (0.77)		2.58 (0.82)
*AQ*					
Total	15.70 (4.98)		18.73 (7.40)		17.58 (6.41)
Attention to detail	5.43 (2.04)		**5.51 (2.42)**	>	**4.32 (2.01)**
Social	**10.28 (4.70)**	<	**13.22 (6.49)**		13.26 (6.51)
Social skills	**1.48 (1.68)**	<	**2.74 (2.49)**		2.61 (2.63)
Communication	1.85 (1.61)		2.49 (2.12)		2.39 (1.73)
Imagination	2.18 (1.52)		2.66 (1.81)		2.87 (1.95)
Attention Switching	4.78 (1.91)		5.33 (2.06)		5.39 (1.92)
*Gold-MSI*					
General ME	**5.68 (0.50)**	>	**4.76 (0.82)**	>	**2.74 (1.07)**
Active engagement	**4.94 (0.81)**	>	**4.02 (1.00)**	>	**2.95 (1.19)**
Formal education	**5.95 (0.56)**	**>**	**4.66 (0.96)**	>	**1.71 (0.68)**
Emotion	**5.88 (0.73)**	>	**5.55(0.78)**	>	**4.95 (1.32)**
Singing	**5.34 (0.83)**	**>**	**4.59 (1.19)**	>	**2.84 (1.26)**
Perception	**6.31 (0.51)**	>	**5.75(0.92)**	>	**4.22 (1.49)**
*PROMS*					
Pitch	**0.27 (0.06)**	>	**0.24 (0.07)**	>	**0.18 (0.06)**
Melody	**0.23 (0.08)**	>	**0.16 (0.10)**	>	**0.07 (0.08)**
Timbre	0.32 (0.08)		0.29 (0.08)		0.26 (0.09)
Rhythm	0.33 (0.08)		**0.32 (0.09)**	>	**0.27 (0.08)**

Note. Descriptive values show mean ratings for the PANAS,^38^ the Big-Five Domains,^39^ and the Gold-MSI.^40^ AQ score were calculated based on Hoekstra et al.^41^ and Baron-Cohen et al.^42^ Comparison signs (“>” or “<”) indicate significant differences. For a full report of statistical details, please refer to OSF. For a detailed description and discussion of the differences between professional musicians and non-musicians, please refer to Nussbaum et al.^14^ Bold entries mark significant differences (*p* <. 05).

#### Emotion classification performance

The mean proportion of correct responses was submitted to an ANOVA with Emotion (Happiness, Pleasure, Fear, and Sadness) and Morph Type (Full, F0, and Timbre) as repeated measures factors and Group (professionals, amateurs, and non-musicians) as a between subjects-factor (see Table 5). For the parallel Bayesian ANOVA, please refer to Table S2 on OSF.

**Table 5:** Results of the 4 × 3 × 3 mixed-effects ANOVA on the mean proportion of correct responses

	df1	df2	*F*	*p*	Ω_p_^2^ [95% CI]	*ε*_HF_
Group	2	163	1.96	0.144	0.01 [0.00, 0.06]	–
Emotion	3	489	130.24	<0.001	0.44 [0.38, 0.49]	–
Morph Type	2	326	1357.80	<0.001	0.89 [0.87, 0.91]	0.829
Group × Emotion	6	489	1.17	0.322	0.00 [0.00, 0.01]	–
Group × Morph Type	4	326	2.14	0.089	0.01 [0.00, 0.04]	0.829
Emotion × Morph Type	6	978	40.95	<0.001	0.20 [0.15, 0.24]	0.875
Group × Emotion × Morph Type	12	978	0.74	0.688	0.00 [0.00, 0.01]	0.865

**Figure 3:**
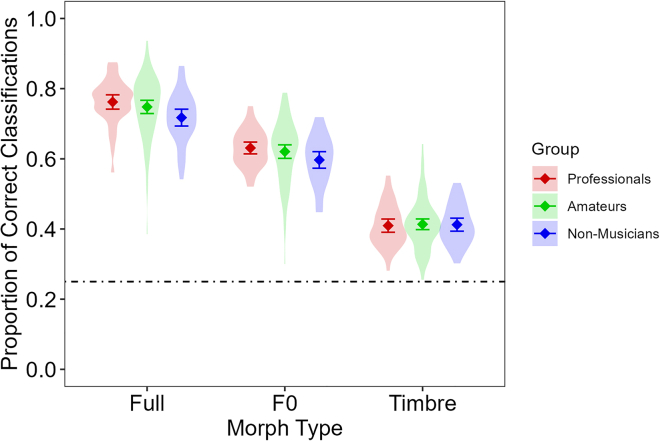
Mean proportion of correct responses per Morph Type separately for professionals, amateurs, and non-musicians Whiskers represent 95% confidence intervals. Violin plots represent variation of individual participants. The dotted line represents guessing rate at 0.25.

The results revealed significant main effects of Emotion and Morph Type, which were qualified by an interaction between Emotion and Morph Type. Please note that these effects were already present in the two datasets that entered the current data (reported in Nussbaum et al.^14^ and part 1 earlier). Because they afford no new information, they are not further detailed here. Crucially, however, we found no main effect involving Group and only a trend for an interaction between Group and Morph Type (Figure 3).

Planned comparisons between professionals and amateurs revealed moderate evidence for the null effect of overall performance (*p* = 0.473, BF_10_ = 0.238), as well as for Full (*p* = 0.322, BF_10_ = 0.286), F0 (*p* = 0.435, BF_10_ = 0.245), and Timbre morphs (*p* = 0.840, BF_10_ = 0.205) separately. Planned comparisons between amateurs and non-musicians revealed inconclusive evidence for overall performance (*p* = 0.107, BF_10_ = 0.546), as well as for Full (*p* = 0.044, BF_10_ = 1.009), and F0 (*p* = 0.105, BF_10_ = 0.576) separately. For Timbre morphs, there was moderate evidence for the null effect (*p* = 0.975, BF_10_ = 0.205). Thus, we found evidence consistent with H5, but inconclusive evidence regarding H4.

## Discussion

In the present study, we shed new light on the link between musicality and VER, by focusing on different subgroups of musicians. In line with our hypotheses, emotion recognition was found to be comparable between both singers and instrumentalists, and also between amateur musicians and professionals. We further replicated the consistent link between sensitivity toward musical patterns and VER, although this was reflected in objective performance measures only, but not in self-rated musicality. In total, these results suggest that the link between musicality and VER is associated with individual differences in auditory sensitivity, which is not tied to a particular type or amount of musical activity. In what follows, we will discuss these findings in more detail.

### Singers versus instrumentalists

For the present study, we recruited a well-powered sample of singers and instrumentalists, which were comparable with regard to personality, socioeconomic background, and objective music perception performance. They only differed in two aspects of self-rated musicality: unsurprisingly, singers scored higher on self-rated singing abilities, and instrumentalists scored higher on self-rated formal education. The latter may be because mastering an instrument to the point where one can play in an ensemble arguably takes more formal training than being able to sing in a choir.

In line with our prediction, the VER performance of singers and instrumentalists did not differ in any condition, as supported by Bayesian analysis with moderate evidence. This finding does not simply represent a general absence of effects. In fact, we replicated the strong and consistent effect of vocal parameters (manipulated via voice morphing) on emotion recognition, with F0 contour being relatively more informative than timbre, across all emotions, while performance was still best in the Full condition. The fact that this pattern was found to be highly comparable for singers and instrumentalists suggests that they use the same acoustic cues to perceive vocal emotions and do so with comparable efficiency. Note that the effects of the F0 and timbre manipulation on emotion recognition also provided a complete replication of the pattern observed in professional musicians and non-musicians in our previous study.^14^ For an even more detailed reflection on the roles of F0 and timbre for emotion recognition, both on a behavioral and neural level (irrespective of musicality), please also refer to Nussbaum et al.^44^

Although this is precisely what we predicted, we want to address two observations from the literature which may seem incompatible with our findings: first, our findings diverge from Thompson et al.,^31^ who found that singing lessons may interfere with vocal emotional processing. However, this study had several methodological limitations including a very small sample size and large drop-out rates. With the present, substantially powered design, we do not see any evidence for a disruptive effect of active singing. This aligns with the broader literature on protective effects of singing on cognitive and socioemotional functioning.^45^^,^^46^ Second, several studies found correlations between singing abilities and emotion recognition.^14^^,^^15^^,^^30^ However, all these studies included both singers and instrumentalists. In fact, it is quite common that instrumentalists have good singing abilities as well, although it is not their preferred form of musical expression. Thus, while we do not argue against a relationship between singing abilities and emotion recognition per se, we lack evidence that emotion recognition is more pronounced in specific groups of musicians, or that this is causally linked to certain musical activities. Instead, this correlation seems to be mediated via natural auditory sensitivity. Note that we failed to find the predicted correlation between self-rated singing abilities and emotion recognition in the present study. This may be attributed to potentially decreased variance in our group of amateurs with regard to self-rated musicality (Gold-MSI). In fact, we did not find any correlations with the Gold-MSI subscales in amateurs, which is in contrast to the pattern we had observed in the sample comprised of professionals and non-musicians.^14^

### Amateurs compared to professional musicians and non-musicians

As predicted, we found evidence that emotion perception performance does not differ between amateur musicians and professionals, further strengthening the notion that the amount of music training is not a major influence on VER abilities.^9^ We also had hypothesized that amateurs would outperform non-musicians, because professionals outperformed them in our previous study.^14^ This comparison yielded inconclusive evidence. We speculate that the present amateur sample was more heterogeneous than the professional one, and as a result, our design may have lacked statistical power to detect potentially small differences between amateurs and non-musicians, despite the substantial sample size. This issue may be resolved in a follow-up study.

In addition to the direct group comparison, we performed a correlational analysis on our sample of amateurs (part 2) which was in parallel to an analysis we had performed previously on professionals and non-musicians.^14^ Importantly, we found a highly similar pattern of correlations between VER and music perception abilities, especially for melody and rhythm. We therefore conclude that individual differences in music perception abilities play an important role, irrespective of the assignment to any (non)-musical group. This is fully in line with the current literature, which consistently emphasizes the role of auditory sensitivity and argues against a causal effect of training or musical activity on VER.^9^^,^^19^

### Conclusion

In the present study, we investigated how emotion perception differs in musical subgroups and compared singers and instrumentalists as well as amateurs and professionals. In line with our prediction, we observed no differences between musical subgroups, suggesting that emotion perception abilities are not primarily tied to the type or amount of musical activity. Instead, we replicated the link between VER and music perception abilities, especially for melodies. This adds a new perspective to the accumulating evidence that the link between musicality and VER is predominantly associated with individual differences in natural auditory sensitivity.

### Limitations of the study

To the best of our knowledge, this study is the first substantially powered comparison between different subgroups of musicians (specifically, singers vs. instrumentalists and professionals vs. amateurs) with respect to VER and therefore closes an open gap in the literature. Nevertheless, the present study has several limitations which deserve consideration. First, our dataset was limited to four emotions expressed through short pseudowords. Future research should examine the extent to which these findings generalize to other types of vocal material. Second, all musicians were socialized in Western music culture and fluent German speakers; therefore, findings may not generalize to other music and language backgrounds.^47^ Note, however, that links between music perception performance and emotion recognition irrespective of formal musical training have been observed in a substantial Portuguese sample of musically trained and untrained participants who varied widely in their musical skill.^15^

What posed a particular challenge in the present study was the recruitment of mutually exclusive groups of musicians. While the distinction between singers and instrumentalists may seem trivial at first glance, it is actually rare that musicians engage in one of these activities only. Thus, for practical reasons, we recruited participants who self-assigned clearly to one form and made sure that their current activity is either one or the other. Yet, some of the singers reported that they used to play an instrument as well. In a similar vein, the distinction between amateurs and professionals is not fully straightforward and may represent a continuum rather than clear-cut categories. Some individuals pursue music as a profession, but without a formal music degree, others vice versa.^48^ Some people transition from amateurs to a professional level later in life.^49^ Thus, while the argument still holds that future research should consider the heterogeneity of musicians, it may be more adequate to pursue variability on a spectrum rather than for distinct groups.

On a practical note, we must acknowledge the technical randomization error. While in the previous study,^14^ stimuli were drawn only once, as intended, the present code allowed full randomization with duplication and omissions of stimuli. While undoubtedly unfortunate, we are nevertheless confident that this error has not affected our results substantially. First, the classification patterns for different Morph Types and Emotions fully replicate our previous study (cf. Figure 2) and we observed highly similar correlations between VER and music perception performance (cf. Table 3). Second, while this issue might have decreased our signal-to-noise ratio, it would not have introduced a specific bias. Thus, we still consider both studies sufficiently comparable.

While the present behavioral data did not reveal differences between musical subgroups, future research may unravel more fine-grained patterns in the brain. Several studies have reported brain differences in auditory and motor processing between amateurs and professionals^50^^,^^51^^,^^52^^,^^53^ and singers and instrumentalists,^54^ but as yet such studies do not provide insights into vocal emotional processing. While we observed differences in electrophysiological responses in professional musicians and non-musicians during VER,^55^^,^^56^ a meaningful fine-grained comparison between singers and instrumentalists, or amateurs and professionals, would arguably require a much bigger sample.

Finally, with respect to the individual dynamics of the tight neural overlap of expression and perception in vocal communication, we hold that it may be worthwhile to focus on another group of vocal experts: professional voice actors or imitators. This is because such experts, unlike singers, are specifically trained to express emotions in the *spoken* voice. In fact, a recent study found that voice actors show enhanced sensitivity for linguistic voice prosody and enhanced neural tracking for voice pitch compared to non-actors.^57^ Vocal acting usually involves deliberate exaggeration of vocal expressions and may therefore require a fine-grained explicit representation of vocal emotions.^58^ While there is research comparing actors and non-actors regarding the expression of emotions,^59^ we do not know how this is mirrored in VER performance and how this group may differ from individuals with singing expertise.

## Resource availability

### Lead contact

Further information and requests for resources and reagents should be directed to and will be fulfilled by the lead contact, Christine Nussbaum (christine.nussbaum@uni-jena.de).

### Materials availability

This study did not generate new unique reagents. Stimulus examples have been deposited at the associated OSF repository (https://osf.io/ascqx/, Cat#101S), in the zip-Folder “stimulus_examples.zip” and are publicly available.

### Data and code availability

• All raw and preprocessed data have been deposited at the associated OSF repository (https://osf.io/ascqx/, Cat#102S) in the zip-folder “Analysis_R_Frequentist.zip” and are publicly available.
• All original code for data analysis has been deposited at the associated OSF repository (https://osf.io/ascqx/, Cat#102S and Cat#103S), in the zip-folder “Analysis_R_Frequentist.zip” and “Analysis_JASP_Bayesian.zip” and is publicly available.
• Supplemental information has been deposited at the associated OSF repository (https://osf.io/ascqx/, Cat#104S), in the document “supplemental information” and are publicly available.

## Acknowledgments

This study was conducted by J.D. in partial fulfilment of the requirements for a master’s thesis. The original voice recordings that served as a basis for creating our stimulus material were provided by Sascha Frühholz. We thank Hannah Strauβ for support with the PROMS. We are grateful to all participants of the study. The abstract was created with Canva (https://www.canva.com/).

## Author contributions

C.N., conceptualization, methodology, software, visualization, formal analysis, writing – original draft, and supervision; J.D., data collection, formal analysis, visualization, and writing – original draft; A.S., methodology, writing – review and editing, and supervision; S.R.S., conceptualization, writing – review and editing, and supervision.

## Declaration of interests

The authors declare no competing interests.

## Declaration of generative AI and AI-assisted technologies in the writing process

No generative AI or AI-assisted technologies were used in the writing process of this manuscript.

## STAR★Methods

### Key resources table

**Table:** 

REAGENT or RESOURCE	SOURCE	IDENTIFIER
**Deposited data**		

a zip-Folder with Stimulus examples: “stimulus_examples.zip”	Associated OSF repository provided by the authors	Cat#101S; OSF: https://osf.io/ascqx/
a zip-Folder containing the frequentist analysis + preprocessed data in R: “Analysis_R_Frequentist.zip”	Associated OSF repository provided by the authors	Cat#102S; OSF: https://osf.io/ascqx/
a zip-Folder containing the Bayesian analysis in JASP: “Analysis_JASP_Bayesian.zip”	Associated OSF repository provided by the authors	Cat#103S; OSF: https://osf.io/ascqx/
a PDF with supplemental information: “Supplemental_Tables_and_Figures.pdf”	Associated OSF repository provided by the authors	Cat#104S; OSF: https://osf.io/ascqx/

**Software and algorithms**		

R Version 4.5.0 (used for statistical analysis)	R Core Team (2025)^60^	https://cran.r-project.org/
JASP Version 0.19.3 (used for statistical analysis)	JASP Team (2025)^61^	https://jasp-stats.org/

### Experimental model and study participant details

#### Participants for part 1 and 2

Note that this is a follow-up to the study reported in Nussbaum et al.^14^ Thus, the stimulus material and the design are almost identical, but we recruited a new sample.

According to our preregistered plan, we aimed at a sample size of 40 singers (20 male, 20 female) and 40 instrumentalists (20 male, 20 female), because in our previous study, this sample size allowed us to reveal medium-sized group effects (d = 0.56 – 0.81) when we compared professional musicians and non-musicians.

Data were collected in a pseudonymized format from June 2023 to January 2024. All participants were aged between 18 and 54 years and fluent German speakers. Participants provided informed consent before completing the experiment and received compensation in the form of 12.50 € or course credit upon completion. The experiment was in line with the ethical guidelines by the American Psychological Association (APA) and approved by the local ethics committee of the Friedrich Schiller University Jena (Reg.-Nr. FSV 19/045).

In total, we collected data from 94 amateur musicians that were divided into singers and instrumentalists. Recruitment criteria specified that participants had to be non-professional musicians (i.e., they held no music-related academic degree or worked professionally as a musician). Singers were required to be currently active in a choir or another singing group, but should not play an instrument actively and regularly (i.e., they must not currently be instrumentalists in an orchestra or a band). Instrumentalists, conversely, were required to be currently active in an orchestra or a band, but they should not engage in singing activities actively and regularly (i.e., they must not currently be in a choir or another singing group).

##### Singers

We recorded data from 48 singers, of which three were excluded (N = 2 had > 5 % trials of omission, N = 1 had technical issues during stimulus playback). Thus, data from 45 singers were analyzed (22 female, 22 male, 1 diverse, aged 18 to 53 years [*M* = 27.02, *SD* = 8.2]). Mean onset age of musical training was 8 years (*SD* = 3.08, 5 – 20 years). Mean duration of musical training was 10 years (*SD* = 6.59, 0 – 25 years). Five participants reported that they never had any formal musical training. Two participants reported that they had occasional tinnitus, but without any subjective impairments in daily life.

##### Instrumentalists

Data from 46 instrumentalists were collected, of which three were excluded. One had technical issues during stimulus playback, one was also active in a choir, one held a master’s degree in music science and was therefore regrouped with the professional musicians (see Participants for part 3). Thus, data from 43 instrumentalists entered analysis (24 female, 18 male, 1 diverse, aged 18 to 54 years [*M* = 28.51, *SD* = 10.64]). Mean onset age of musical training was 7 years (*SD* = 2.27, 4 – 14 years). Mean duration of musical training was 14 years (*SD* = 10.00, 0 – 44 years). Four participants reported that they never had any formal musical training, thus were autodidacts. For more details, please refer to the supplemental sample information in the document “supplemental information” in the associated OSF repository (https://osf.io/ascqx/): Table S16 for musical background, Table S17 for socioeconomic background.

#### Participants for part 3

For this analysis, we collapsed all participants from part 1 into the group of amateur musicians and compared it to the groups of professional musicians and non-musicians reported in Nussbaum et al.^14^ Note that we added one participant to the professional group, because he held a master’s degree in music (see Participants for part 1 and 2), so numbers slightly diverge from the original publication. All professional musicians reported to have a music-related academic degree or a non-academic music qualification. Non-musicians played no instrument or engaged in any other musical activities. For a more detailed description, please refer to Nussbaum et al.^14^

In total, we analyzed data from 40 professional musicians (20 male, 20 female, aged 20 to 42 years [*M* = 29.6; *SD* = 5.58]), 38 non-musicians (18 male, 20 female, aged 19 to 48 years [*M* = 30.5; *SD* = 6.54]) and 88 amateurs (40 male, 46 female, 2 diverse, aged 18 to 54 years [*M* = 27.8; *SD* = 9.44]).

### Method details

#### Stimulus material

As stimulus material, we used parameter-specific voice morphs that express emotional information either through the fundamental frequency contour only (F0), through timbre only (Tbr) or through a combination of both (Full).

For voice morphing, we selected original audio recordings from a database of vocal actor portrayals, comprised of pseudowords (/molen/, /loman/, /belam/) uttered by eight speakers (four male, four female) with expressions of happiness, pleasure, fear, and sadness. We specifically opted for two positive and two negative emotions of different intensities, to balance both valence and arousal. A prior validation study with 20 raters confirmed that the two positive and two negative emotions had different degrees of emotional intensity (happiness > pleasure, t(19)=9.57, p < 0.001 and fear > sadness, t(19)=6.58, p < 0.001).

To synthesize the parameter-specific emotional voice morphs, we created morphing trajectories between each emotion and an emotional average of the same speaker and pseudoword, using the Tandem-STRAIGHT software.^62^^,^^63^ The averages had been created previously by blending all emotions together and were thus assumed to be uninformative and unbiased with respect to the four emotions of interest. Tandem-STRAIGHT enables voice morphing via weighted interpolation of five independent parameters: (1) F0-contour, (2) timing, (3) spectrum-level, (4) aperiodicity, and (5) spectral frequency; the latter three are summarized as timbre.

**Figure 4:**
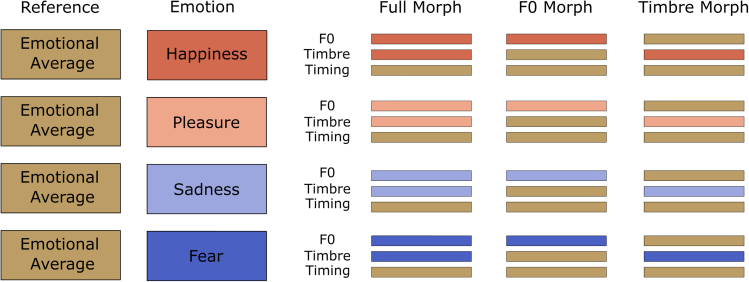
Morphing matrix for stimuli with averaged voices as reference Figure reprinted from Nussbaum et al.,^14^ Figure 2, page 6.

We created three types of morphed stimuli (see Figure 4). Full-Morphs were stimuli with all parameters taken from the emotional version (corresponding to 100% from the emotion and 0% from average), except for the timing parameter, which was taken from the average (corresponding to 0% emotion and 100% average). F0-Morphs were stimuli with the F0-contour taken from the emotion, but timbre and timing were taken from the average. Timbre-Morphs were stimuli with all timbre parameters taken from the emotion, but F0 and timing from the average. Note that the timing was kept constant in all conditions to allow a pure comparison of F0 vs. timbre. Furthermore, we kept all average stimuli as a further ambiguous reference category. In total, this resulted in 8 (speakers) x 3 (pseudowords) x 4 (emotions) x 3 (morphing conditions) + 24 average (8 speakers x 3 pseudowords) = 312 stimuli (duration M = 780 ms, range 620 to 967 ms, SD = 98 ms). Using PRAAT,^64^ we normalized all stimuli to a root-mean-square of 70 dB SPL.

For a more detailed description of the stimulus creation, see Nussbaum et al.^14^ and Kawahara and Skuk, 2018.^65^ For a summary of acoustic characteristics, see Tables S18 and S19 in the document “supplemental information” in the associated OSF repository (https://osf.io/ascqx/).

#### Design

Data were collected online via PsyToolkit,^66^^,^^67^ but after completion of the study all participants met with the experimenter for a short personal debriefing. This was done to increase commitment and conscientiousness for the experiment.

Participants were required to ensure a quiet environment for the duration of the study and use a computer with a physical keyboard and headphones. Prior to the listening tasks, participants could adjust their sound settings to a comfortable sound pressure level.

First, participants entered demographic information, including age, sex, native language, profession, and potential hearing impairments such as tinnitus. They then completed an emotion classification experiment, a test on music perception and several questionnaires on musicality, personality and socioeconomic background. Mean duration of the whole online study was about 75 minutes.

##### Emotion classification experiment

In the experiment, participants classified vocal emotions as happiness, pleasure, fear, or sadness. Each trial started with a green fixation cross presented for 500 ms. Then the sound was played while a loudspeaker symbol was shown on the screen. Subsequently, a response screen showed the emotion labels and participants could enter their response within a 5000 ms time window starting from voice offset. Responses were entered with the left and right index and middle fingers, with random mapping of response keys to emotion categories for each participant, out of four possible key mappings (see Tables S20 and S21 in the document “supplemental information” in the associated OSF repository). In case of a response omission, the final trial slide (500 ms) prompted participants to respond faster; otherwise, the screen turned black. Then the next trial started.

The 312 stimuli were presented in randomized order in six blocks of 52 trials each, with self-paced breaks in between. Beforehand, participants completed eight practice trials with different stimuli. The experiment was about 25 minutes long. Unfortunately, due to a software error, randomization was sampled with replacements, so that some stimuli were drawn repeatedly and others were omitted. This was in contrast to our previous study, where randomization was sampled without replacement so that each stimulus was drawn exactly once.

##### Profile of Music Perception Skills (PROMS)

To measure music perception skills, we used a modular version of the Profile of Music Perception Skills,^68^^,^^69^ comprised of the four subtests „Melody“, „Pitch“, „Timbre”, and „Rhythm“, which we considered most informative for the present research question. Participants completed 18 items per subtest, always preceded by one practice trial. Each trial, participants heard a reference stimulus twice followed by a target stimulus. Then, they indicated whether reference and target were the same or different via a 5-point Likert scale with the labels “definitely same”, “maybe same”, “don’t know”, “maybe different”, and “definitely different”. The duration of the PROMS was about 20 minutes.

##### Questionnaires

After the PROMS, participants completed several questionnaires: the German Version of the Autism Quotient Questionnaire, AQ,^42^^,^^70^ a 30-item Personality Inventory measuring the Big-Five domains,^39^ the Goldsmiths Musical Sophistication Index, Gold-MSI,^40^ to assess the participants’ degree of self-reported musical skills, additional questions concerning music experience and musical engagement, their socioeconomic background, and the 20-item version of the Positive-Affect-Negative-Affect-Scale, PANAS.^38^^,^^71^

### Quantification and statistical analysis

In line with our preregistered plan, we collapsed data across speakers and pseudowords for analysis. Further, data on emotional averages were excluded because they were not relevant for our hypotheses. Response omissions (∼1 %) were treated as errors and participants with more than 5% of such omissions excluded from data analysis. Analyses of Variance (ANOVAs) and correlational analyses were performed using R Version 4.5.0.^60^ Post-hoc tests were Benjamini-Hochberg corrected where appropriate.^43^ A p-value < 0.05 was considered significant.

We complemented these classical frequentist analyses with a Bayesian approach, which – in contrast to null hypothesis significance testing - allows a quantification of evidence for null findings.^72^ These analyses were conducted in JASP Version 0.19.3^61^ using default priors, which have been considered appropriate for testing null hypotheses based on similar sample sizes.^19^^,^^73^ Further, we ensured that our Bayesian inference did not depend critically on the choice of priors by running robustness checks (see data analysis files on OSF). We report the Bayes factor (BF_10_) as an indicator for the likelihood of the null and alternative hypothesis given the observed data. BF_10_ > 1 indicate larger evidence for the alternative hypothesis, BF_10_ < 1 indicate larger evidence for the null hypothesis. For example, a BF_10_ = 3 means that the alternative hypothesis is three times more likely than the null hypothesis, whereas the reciprocal BF_10_ = .33 means that the null hypothesis is three times more likely than the alternative. Following the guidelines by Jarosz et al. 2014,^74^ we consider values of BF_10_ = 1-3 (.1-.33) as anecdotal, BF_10_ = 3-10 (.33-.10) as moderate, BF_10_ = 10-30 (.10-.03) as strong, BF_10_ = 30-100 (.03-.01) as very strong and BF_10_ > 100 (< .01) as decisive evidence for the alternative hypothesis and the reciprocal values in parentheses as respective evidence for the null hypothesis.

In alignment with the approach by Nussbaum et al.,^14^ we first recoded responses in the PROMS from 1 to 0 in 0.25 steps starting with the “definitely” correct option down to the “definitely” incorrect option (thus, “don’t know” was always coded with 0.5). For the final measure, we then subtracted 0.5, so that a positive score indicates that participants were more correct/confident, a negative score indicates more incorrect/uncertain ratings, and a score of zero indicates responses at chance level. For statistical analyses, we used the averaged performance across trials for each subtest.

For part 2, we calculated Spearman correlations between vocal emotion recognition performance and both the PROMS music perception performance and the Gold-MSI self-rated musicality. P-values were adjusted for multiple comparisons using the Benjamini-Hochberg correction.^43^ Following our pre-registered plan, all correlations were controlled for formal musical education as well, but we found that this made no difference to the observed patterns.

For part 3, we focused our analysis on the comparison of amateurs with the other two groups, because the comparison of professional musicians and non-musicians is reported in Nussbaum et al.^14^

### Additional resources

We specified how we determined our sample size, all data exclusions, all manipulations, and all measures in the associated preregistration (https://doi.org/10.17605/OSF.IO/76PV5), published on June 9, 2023, thus before we started data collection on June 12, 2023. Please note that the numbering and wording of hypotheses was slightly modified from the preregistration to increase clarity, while not affecting their content. Preprocessed data, analysis scripts and supplemental information can be found in the associated OSF repository (https://osf.io/ascqx/).
