# Supplementary material for: Vocal emotion recognition: A comparison of singers and instrumentalists, amateurs and professionals
Source: iScience. 2025 Nov 17;28(12):114089. doi: 10.1016/j.isci.2025.114089 (PMC12756571; doi:10.1016/j.isci.2025.114089)
Supplement: Document S1. Figures S1–S5 and Tables S1–S21 [file mmc1.pdf]

iScience, Volume *28*

Supplemental information

Vocal emotion recognition: A comparison of singers and instrumentalists, amateurs and professionals

Christine Nussbaum, Jessica Dethloff, Annett Schirmer, and Stefan R. Schweinberger

Table S1 – Bayesian Repeated Measures ANOVA (Singers vs. Instrumentalists)

Models	P(M)	P(M|data)	BF_M	BF_{10}	error %
Emotion + MType + Emotion ✻ MType	0.053	0.808	75.897	1.000	
Emotion + MType + Group + Emotion ✻ MType	0.053	0.130	2.701	0.161	2.042
Emotion + MType + Group + Emotion ✻ MType + Emotion ✻ Group	0.053	0.054	1.036	0.067	3.630
Emotion + MType + Group + Emotion ✻ MType + MType ✻ Group	0.053	0.005	0.088	0.006	3.103
Emotion + MType + Group + Emotion ✻ MType + Emotion ✻ Group + MType ✻ Group	0.053	0.002	0.033	0.002	2.468
Emotion + MType + Group + Emotion ✻ MType + Emotion ✻ Group + MType ✻ Group + Emotion ✻ MType ✻ Group	0.053	1.600×10^{-4}	0.003	1.979×10^{-4}	6.094
Emotion + MType	0.053	4.463×10^{-24}	8.034×10^{-23}	5.522×10^{-24}	2.680
Emotion + MType + Group	0.053	6.564×10^{-25}	1.182×10^{-23}	8.121×10^{-25}	2.251
Emotion + MType + Group + Emotion ✻ Group	0.053	2.308×10^{-25}	4.155×10^{-24}	2.856×10^{-25}	3.079
Emotion + MType + Group + MType ✻ Group	0.053	2.684×10^{-26}	4.832×10^{-25}	3.321×10^{-26}	14.133

Note. All models include subject, and random slopes for all repeated measures factors.
Note. Showing the best 10 out of 19 models.

Note. For interpretation of the output, refer to van den Bergh et al. (2020) [1]

Table S2 – Bayesian Repeated Measures ANOVA (Professionals vs. Amateurs vs. Non-Musicians)

Models	P(M)	P(M|data)	BF_M	BF_{10}	error %
Emotion + MType + Emotion ✻ MType	0.053	0.870	120.156	1.000	
Emotion + MType + Group + Emotion ✻ MType	0.053	0.122	2.495	0.140	4.243
Emotion + MType + Group + Emotion ✻ MType + Emotion ✻ Group	0.053	0.005	0.082	0.005	2.941
Emotion + MType + Group + Emotion ✻ MType + MType ✻ Group	0.053	0.004	0.070	0.004	2.480
Emotion + MType + Group + Emotion ✻ MType + Emotion ✻ Group + MType ✻ Group	0.053	1.492×10^{-4}	0.003	1.715×10^{-4}	2.922
Emotion + MType + Group + Emotion ✻ MType + Emotion ✻ Group + MType ✻ Group + Emotion ✻ MType ✻ Group	0.053	2.669×10^{-7}	4.804×10^{-6}	3.069×10^{-7}	2.457
Emotion + MType	0.053	1.444×10^{-53}	2.599×10^{-52}	1.660×10^{-53}	2.095
Emotion + MType + Group	0.053	1.705×10^{-54}	3.069×10^{-53}	1.961×10^{-54}	5.388
Emotion + MType + Group + Emotion ✻ Group	0.053	4.577×10^{-56}	8.239×10^{-55}	5.263×10^{-56}	3.204
Emotion + MType + Group + MType ✻ Group	0.053	3.260×10^{-56}	5.868×10^{-55}	3.748×10^{-56}	5.269

Note. All models include subject, and random slopes for all repeated measures factors.
Note. Showing the best 10 out of 19 models.

Note. For interpretation of the output, refer to van den Bergh et al. (2020) [1]

Figure S1

Confusion data for each Emotion for the three Morph Types – Singers and Instrumentalists

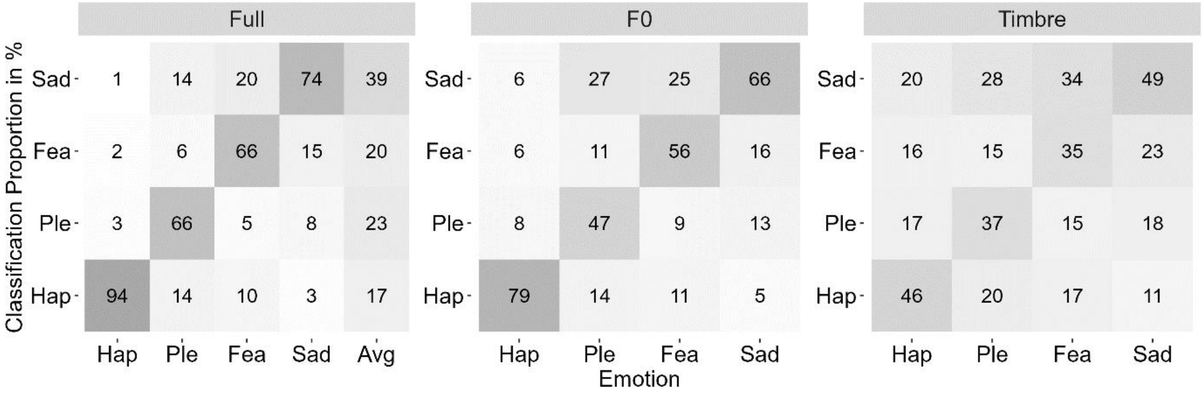

Note. Numbers represent the proportion of classification responses per Emotion and Morph Type, averaged across musicians. Hap = happiness, Ple = pleasure, Fea = fear, Sad = sadness, Avg = average.

Figure S2

Confusion data for each Emotion for the three Morph Types – Singers only

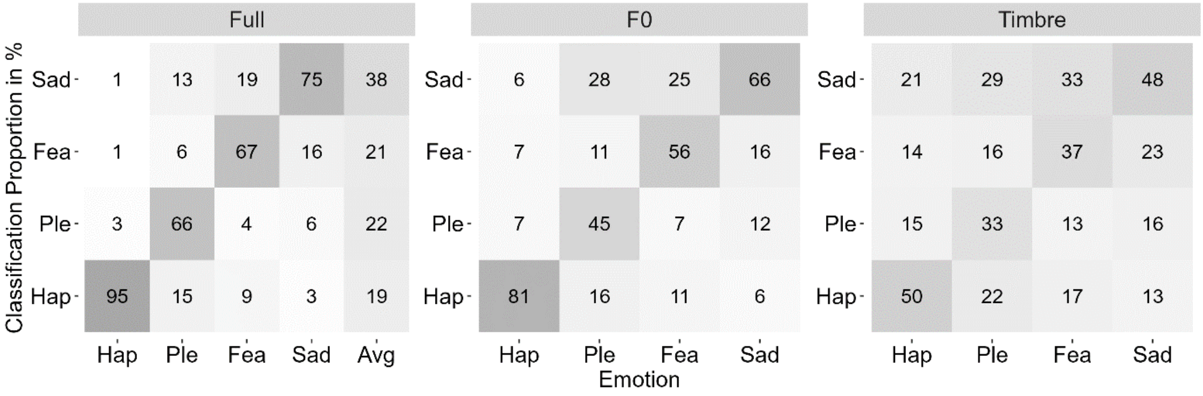

Note. *Numbers represent the proportion of classification responses per Emotion and Morph Type, averaged across non-musicians. Hap = happiness, Ple = pleasure, Fea = fear, Sad = sadness, Avg = average.*

Figure S3

Confusion data for each Emotion for the three Morph Types – Instrumentalists only

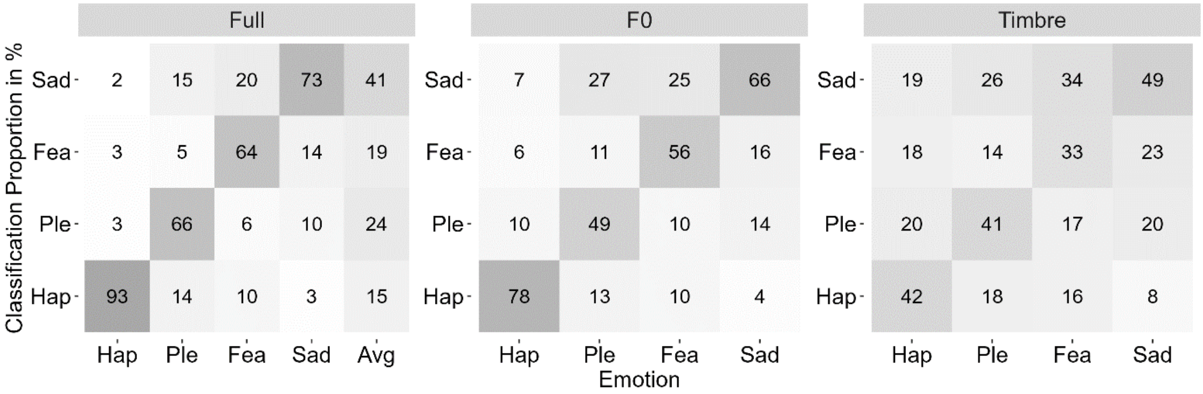

Note. *Numbers represent the proportion of classification responses per Emotion and Morph Type, averaged across non-musicians. Hap = happiness, Ple = pleasure, Fea = fear, Sad = sadness, Avg = average.*

Figure S4

Correlation between Emotion Classification Performance and Music Perception Abilities (PROMS)

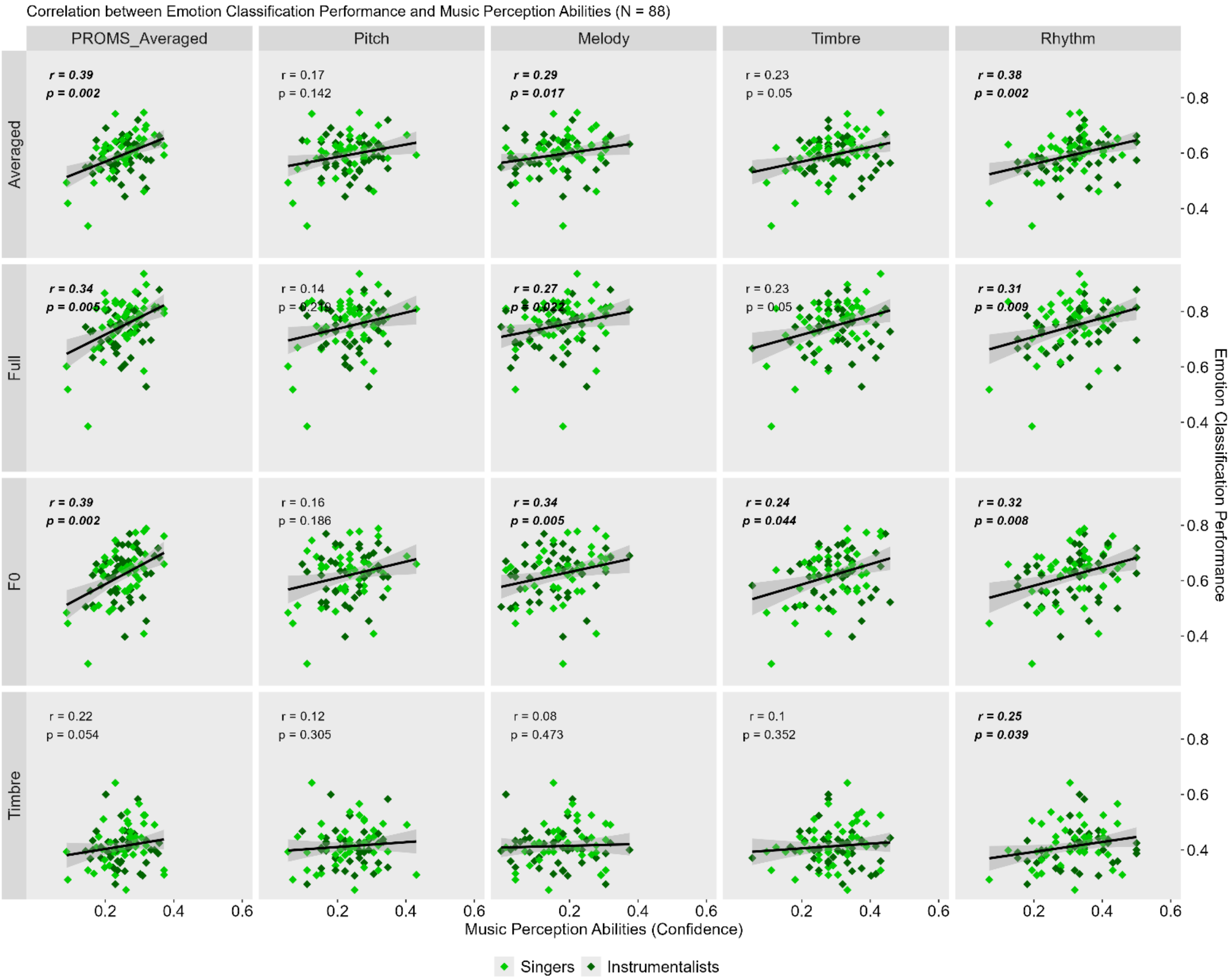

Note. Correlations are not controlled for formal musical education. The x-axis shows the different subtests of the PROMS (Pitch, Melody, Timbre, and Rhythm) as well as the averaged performance across all subtests (PROMS_Averaged). The y-axis shows the vocal emotion classification performance separately for each Morph Type (Full, F0 and Timbre) and averaged across Morph Types (Averaged). Correlations are not controlled for formal musical education. p-values were adjusted for multiple comparisons using the Benjamini-Hochberg correction [2]

Figure S5

Correlation between Emotion Classification Performance and self-rated Music Skills (GOLD-MSI)

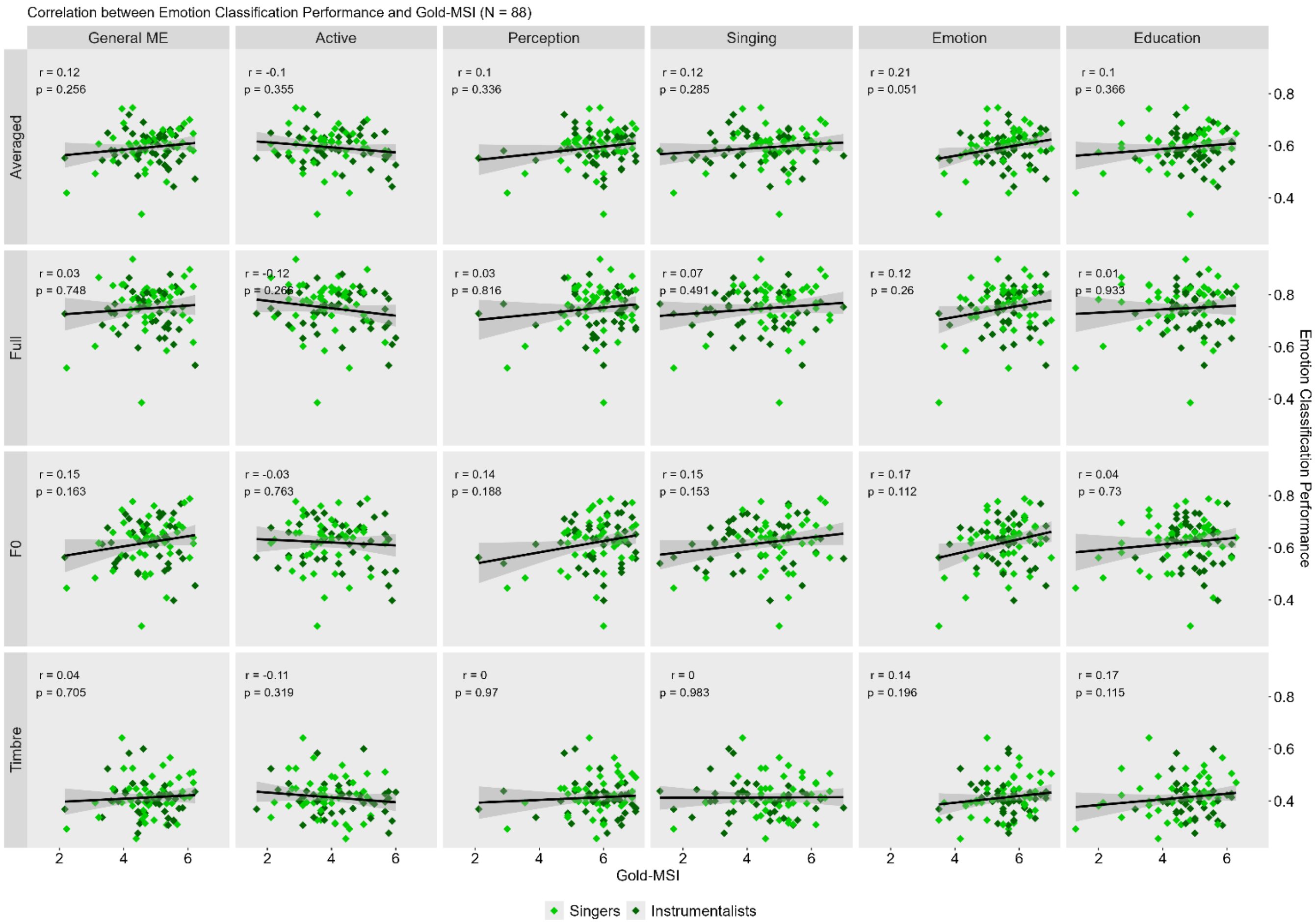

Note. The x-axis shows the different subscores of the Gold-MSI (Active, Perception, Singing, Emotion, and Education) as well as the General Music Education score (General ME). The y-axis shows the vocal emotion classification performance separately for each Morph Type (Full, F0 and Timbre) and averaged across Morph Types (Averaged). Correlations are not controlled for formal musical education. p-values were adjusted for multiple comparisons using the Benjamini-Hochberg correction
2

Table S3 – PROMS and VER, correlations

	PROMSAvg	Pitch	Melody	Timbre	Rhythm
VERAvg	**.39 (.002)**	.17 (.142)	**.29 (.017)**	.23 (.050)	**.38 (.002)**
Full-Morphs	**.34 (.005)**	.14 (.219)	**.27 (.022)**	.23 (.050)	**.31 (.009)**
F0-Morphs	**.39 (.002)**	.16 (.186)	**.34 (.005)**	**.24 (.044)**	**.32 (.008)**
Timbre-Morphs	.22 (.054)	.12 (.305)	.08 (.473)	.10 (.352)	**.25 (.039)**

Note. VER = Vocal Emotion Recognition performance. p-values of Tables S9 – S12 were adjusted for multiple comparisons using the Benjamini-Hochberg correction [2]. Table S3 is identical with Table 3 from the manuscript.

Table S4 – PROMS and VER, controlled for musical education

	PROMSAvg	Pitch	Melody	Timbre	Rhythm
VERAvg	**.38 (.003)**	.15 (.204)	**.27 (.023)**	.22 (.066)	**.36 (.003)**
Full-Morphs	**.35 (.005)**	.14 (.212)	**.28 (.023)**	.23 (.058)	**.32 (.008)**
F0-Morphs	**.39 (.003)**	.15 (.204)	**.34 (.006)**	.24 (.053)	**.32 (.008)**
Timbre-Morphs	.18 (.124)	.08 (.503)	.05 (.673)	.08 (.499)	.22 (.062)

Note. VER = Vocal Emotion Recognition performance.

Table S5 – MSI and VER, correlation*s*

	General Sophistication	Active Engagement	Musical Training	Emotions	Singing Abilities	Perceptual Abilities
VER_{Avg}	.12 (.256)	-.1 (.355)	.10 (.366)	.21 (.051)	.12 (.285)	.10 (.336)
Full-Morphs	.03 (.748)	-.12 (.266)	.01 (.933)	.12 (.260)	.07 (.491)	.03 (.816)
F0-Morphs	.15 (.163)	-.03 (.763)	.04 (.730)	.17 (.112)	.15 (.153)	.14 (.188)
Timbre-Morphs	.04 (.705)	-.11 (.319)	.17 (.115)	.14 (.196)	.00 (.983)	.00 (.970)

Note. VER = Vocal Emotion Recognition performance.

Table S6 – MSI and VER, controlled for musical training

	General Sophistication	Active Engagement	Emotions	Singing Abilities	Perceptual Abilities
VER_{Avg}	.08 (.627)	-.16 (.509)	.19 (.509)	.09 (.627)	.07 (.627)
Full-Morphs	.04 (.769)	-.14 (.509)	.13 (.534)	.07 (.627)	.02 (.829)
F0-Morphs	.16 (.509)	-.06 (.711)	.17 (.509)	.15 (.509)	.14 (.509)
Timbre-Morphs	-.09 (.627)	-.21 (.509)	.08 (.627)	-.05 (.731)	-.07 (.627)

Note. VER = Vocal Emotion Recognition performance.

Table S7

Post-hoc tests on the AQ for professionals vs. non-musicians

	**Pro-fessionals**	**Non-musicians**					
	M (SD)	*M (SD)*	*t*	*df*[a]	*p*	*Cohens d*	
AQ							
Total	15.7 (4.98)	17.58 (6.41)	-1.44	69.83	.154	-0.34 [-0.82, 0.13]	
Attention to Detail	**5.43 (2.04)**	**4.32 (2.01)**	2.42	75.87	.018	0.56 [0.09, 1.01]	*
Social	**10.28 (4.70)**	**13.26 (6.51)**	-2.32	67.08	.024	-0.57 [-1.05, -0.08]	*
Social Skills	**1.48 (1.68)**	**2.61 (2.63)**	-2.25	62.40	.028	-0.57 [-1.07, -0.06]	*
Communication	1.85 (1.61)	2.39 (1.73)	-1.44	74.83	.155	-0.33 [-0.79, 0.13]	
Imagination	2.18 (1.52)	2.87 (1.95)	-1.75	69.92	.085	-0.42 [-0.89, 0.06]	
Attention Switching	4.78 (1.91)	5.39 (1.92)	-1.43	75.75	.158	-0.33 [-0.78, 0.13]	

[a] *Note that original degrees of freedom were 76 but were corrected due to unequal variance.*

Table S8

Post-hoc tests on the AQ for professionals vs. amateurs

	**Pro-fessionals**	**Amateurs**					
	M (SD)	*M (SD)*	*t*	*df*[a]	*p*	*Cohens d*	
AQ							
Total	**15.7 (4.98)**	**18.73 (7.40)**	-2.72	107.7	.008	-0.52 [-0.91, -0.14]	**
Attention to Detail	5.43 (2.04)	5.51 (2.42)	-0.21	88.61	.835	-0.04 [-0.46, 0.37]	
Social	**10.28 (4.70)**	**13.22 (6.49)**	-2.90	101.8	.005	-0.57 [-0.97, -0.18]	**
Social Skills	**1.48 (1.68)**	**2.74 (2.49)**	-3.36	107.8	.001	-0.65 [-1.03, -0.26]	**
Communication	1.85 (1.61)	2.49 (2.12)	-1.88	97.42	.063	-0.38 [-0.78, 0.02]	
Imagination	2.18 (1.52)	2.66 (1.81)	-1.57	89.18	.120	-0.33 [-0.75, 0.09]	
Attention Switching	4.78 (1.91)	5.33 (2.06)	-1.48	80.83	.142	-0.33 [-0.77, 0.11]	

[a] *Note that original degrees of freedom were 126 but were corrected due to unequal variance.*

Table S9

Post-hoc tests on the AQ for amateurs vs. non-musicians

	**Amateurs**	**Non-musicians**					
	M (SD)	*M (SD)*	*t*	*df*[a]	*p*	*Cohens d*	
AQ							
Total	18.73 (7.40)	17.58 (6.41)	-0.88	80.44	.382	-0.2 [-0.63, 0.24]	
Attention to Detail	**5.51 (2.42)**	**4.32 (2.01)**	-2.87	83.53	.005	-0.63 [-1.07, -0.19]	**
Social	13.22 (6.49)	13.26 (6.51)	0.04	70.10	.970	0.01 [-0.46, 0.48]	
Social Skills	2.74 (2.49)	2.61 (2.63)	-0.27	67.11	.791	-0.06 [-0.54, 0.41]	
Communication	2.49 (2.12)	2.39 (1.73)	-0.26	85.06	.795	-0.06 [-0.48, 0.37]	
Imagination	2.66 (1.81)	2.87 (1.95)	0.57	65.93	.574	0.14 [-0.34, 0.62]	
Attention Switching	5.33 (2.06)	5.39 (1.92)	0.17	74.89	.865	0.04 [-0.41, 0.49]	

[a] *Note that original degrees of freedom were 124 but were corrected due to unequal variance.*

Table S10

Post-hoc tests on the Gold-MSI for professionals vs. non-musicians

	**Pro-fessionals**	**Non-musicians**					
	M (SD)	*M (SD)*	*t*	*df*[a]	*p*	*Cohens d*	
Gold-MSI							
General ME	**5.68 (0.50)**	**2.74 (1.07)**	15.45	51.63	<.001	4.30 [3.30, 5.28]	***
Active Engagement	**4.94 (0.81)**	**2.95 (1.19)**	8.55	64.53	<.001	2.13 [1.51, 2.73]	***
Formal Education	**5.95 (0.56)**	**1.71 (0.68)**	30.10	71.67	<.001	7.11 [5.85, 8.36]	***
Emotion	**5.88 (0.73)**	**4.95 (1.32)**	3.79	56.87	<.001	1.00 [0.45, 1.55]	***
Singing	**5.34 (0.83)**	**2.84 (1.26)**	10.3	63.49	<.001	2.59 [1.91, 3.25]	***
Perception	**6.31 (0.51)**	**4.22 (1.49)**	8.19	45.10	<.001	2.44 [1.66, 3.20]	***

[a] *Note that original degrees of freedom were 76 but were corrected due to unequal variance.*

Table S11

Post-hoc tests on the Gold-MSI for professionals vs. amateurs

	**Pro-fessionals**	**Amateurs**					
	M (SD)	*M (SD)*	*t*	*df*[a]	*p*	*Cohens d*	
Gold-MSI							
General ME	**5.68 (0.50)**	**4.76 (0.82)**	7.80	116.1	<.001	1.45 [1.04, 1.85]	***
Active Engagement	**4.94 (0.81)**	**4.02 (1.00)**	5.54	91.98	<.001	1.16 [0.71, 1.59]	***
Formal Education	**5.95 (0.56)**	**4.66 (0.96)**	9.54	118.5	<.001	1.75 [1.33, 2.17]	***
Emotion	**5.88 (0.73)**	**5.55 (0.78)**	2.29	80.76	.025	0.51 [0.06, 0.95]	*
Singing	**5.34 (0.83)**	**4.59 (1.19)**	4.08	105	<.001	0.80 [0.40, 1.19]	***
Perception	**6.31 (0.51)**	**5.75 (0.92)**	4.42	121.3	<.001	0.80 [0.43, 1.17]	***

[a] *Note that original degrees of freedom were 126 but were corrected due to unequal variance.*

Table S12

Post-hoc tests on the Gold-MSI for amateurs vs. non-musicians

	**Amateurs**	**Non-musicians**					
	M (SD)	*M (SD)*	*t*	*df*[a]	*p*	*Cohens d*	
Gold-MSI							
General ME	**4.76 (0.82)**	**2.74 (1.07)**	-10.41	56.76	<.001	-2.76 [-3.48, -2.03]	***
Active Engagement	**4.02 (1.00)**	**2.95 (1.19)**	-4.81	60.26	<.001	-1.24 [-1.79, -0.68]	***
Formal Education	**4.66 (0.96)**	**1.71 (0.68)**	-19.67	97.53	<.001	-3.98 [-4.66, -3.29]	***
Emotion	**5.55 (0.78)**	**4.95 (1.32)**	-2.59	48.55	.013	-0.74 [-1.32, -0.16]	*
Singing	**4.59 (1.19)**	**2.84 (1.26)**	-7.30	66.78	<.001	-1.79 [-2.35, -1.22]	***
Perception	**5.75 (0.92)**	**4.22 (1.49)**	-5.85	49.64	<.001	-1.66 [-2.30, -1.01]	***

[a] *Note that original degrees of freedom were 124 but were corrected due to unequal variance.*

Table S13

PROMS post-hoc tests for professionals vs. non-musicians

	**Pro-fessionals**	**Non-musicians**					
	M (SD)	*M (SD)*	*t*	*df*[a]	*p*	*Cohens d*	
PROMS							
Pitch	**0.27 (0.06)**	**0.18 (0.06)**	6.25	75.77	<.001	1.43 [0.93, 1.94]	***
Melody	**0.23 (0.08)**	**0.07 (0.08)**	9.42	75.95	<.001	2.16 [1.59, 2.72]	***
Timbre	**0.32 (0.08)**	**0.26 (0.09)**	2.99	73.64	.004	0.70 [0.22, 1.16]	**
Rhythm	**0.33 (0.08)**	**0.27 (0.08)**	3.52	75.96	<.001	0.81 [0.34, 1.27]	***

[a] *Note that original degrees of freedom were 76 but were corrected due to unequal variance.*

Table S14

PROMS post-hoc tests for professionals vs. amateurs

	**Pro-fessionals**	**Amateurs**					
	M (SD)	*M (SD)*	*t*	*df*[a]	*p*	*Cohens d*	
PROMS							
Pitch	**0.27 (0.06)**	**0.24 (0.07)**	2.57	87.32	.012	0.55 [0.12, 0.98]	*
Melody	**0.23 (0.08)**	**0.16 (0.10)**	4.42	95.24	<.001	0.91 [0.48, 1.33]	***
Timbre	0.32 (0.08)	0.29 (0.08)	1.72	74.69	.090	0.40 [-0.06, 0.85]	
Rhythm	0.33 (0.08)	0.32 (0.09)	0.80	84.27	.425	0.17 [-0.25, 0.60]	

[a] *Note that original degrees of freedom were 126 but were corrected due to unequal variance.*

Table S15

PROMS post-hoc tests for amateurs vs. non-musicians

	**Amateurs**	**Non-musicians**					
	M (SD)	*M (SD)*	*t*	*df*[a]	*p*	*Cohens d*	
PROMS							
Pitch	**0.24 (0.07)**	**0.18 (0.06)**	-4.39	81.21	<.001	-0.97 [-1.43, -0.51]	***
Melody	**0.16 (0.10)**	**0.07 (0.08)**	-5.65	91.34	<.001	-1.18 [-1.62, -0.74]	***
Timbre	0.29 (0.08)	0.26 (0.09)	-1.88	62.25	.064	-0.48 [-0.98, 0.03]	
Rhythm	**0.32 (0.09)**	**0.27 (0.08)**	-3.16	80.84	.002	-0.70 [-1.15, -0.25]	***

[a] *Note that original degrees of freedom were 124 but were corrected due to unequal variance.*

1. Supplemental Sample Information

Table S16

List of reported instruments by amateurs (singers and instrumentalists)

Singers		Instrumentalists	
Gesang *(singing)* / Chor *(choir)*	35	Violine *(violin)*	10
+ Klavier *(piano)*	3	Posaune (*trombone*)	6
+ Violine (*violin*)	2	Cello (*cello*)	5
+ Gitarre *(guitar)*	2	Klarinette (*clarinet*)	4
+ Bass (*bass*)	1	Trompete (*trumpet*)	4
+ Klavier *(piano)* and Cello *(cello)*	1	Waldhorn (*horn*)	2
+ Trompete (*trumpet*)	1	Bratsche (*Viola*)	2
		Schlagzeug (*drums*)	2
		Bariton (*bariton*)	1
		Bass (*bass*)	1
		Fagott (bassoon)	1
		Gitarre *(guitar)*	1
		Klavier *(piano)*	1
		Querflöte (*flute*)	1
		Tuba (*tuba*)	1
		Saxophon (*saxophone*)	1

Table S17

Socioeconomic background of amateurs (singers and instrumentalists)

**Income (in €)**			**Education**			**Degree**		
	**S**	**I**		**S**	**I**		**S**	**I**
<1750	5	4	keine *(none)*	0	0	keine *(none)*	0	1
1750-2500	6	7	Schüler *(pupil)*	0	1	Schüler *(pupil)*	0	0
2500-3500	11	10	Hauptschule *(secondary school)*	0	0	In Ausbildung *(under training)*	15	17
3500-5000	17	9	Mittelschule *(secondary school)*	0	0	Lehre *(traineeship)*	0	3
>5000	6	13	Fachschule *(technical college)*	1	1	Fachschule *(technical college)*	1	0
			Abitur *(A-levels)*	44	41	Meister *(master craftsmen)*	0	0
						Bachelor *(Bachelor)*	10	4
						Fachhochschulabschluss *(polytechnic degree)*	1	2
						Master/Diplom *(Master/Diploma)*	14	10
						Promotion *(PhD)*	4	6
$\chi 2 = 5.23$, df = 4, $p = 0.264$			$\chi 2 = 1.06$, df = 2, $p = 0.588$			$\chi 2 = 9.06$, df = 7, $p = 0.249$		

Note. This table presents the number of individuals belonging to different income, education, and degree categories. We tested group differences between singers (S) and instrumentalists (I) using a Chi-square test and show the results in the last line of this table. Please note that the response options "Education" (i.e. the type of school) and "Degree" (i.e. the highest professional qualification) were tailored to the German educational system and are therefore difficult to translate Further, please note that "Fachschule" and "Abitur" are similar as they both enable a person to pursue a university degree (with a few more constrains for a "Fachschule" degree). We therefore consider the trend observed for the "Education" factor merely as an artefact of the response format. S = Singers, I = Instrumentalists

2. Supplemental Stimulus Information

Table S18

Summary of the acoustic characteristics of female voice morphs separately for each Emotion and Morph Type

	MType	F0 Mean	F0 SD	F0 Glide	FormDisp	HNR
Happiness						
	Full	348	98	-112	993	19
	F0	348	98	-112	1096	20
	Timbre	247	25	-37	981	19
Pleasure						
	Full	185	21	-32	1131	19
	F0	185	21	-32	1094	19
	Timbre	247	25	-37	1122	20
Fear						
	Full	288	30	28	1112	21
	F0	288	30	28	1093	21
	Timbre	247	25	-37	1120	21
Sadness						
	Full	219	19	-39	1090	22
	F0	219	19	-39	1097	21
	Timbre	247	25	-37	1085	22
Average						
	Full	247	25	-39	1094	22

Note. All acoustical parameters were adapted from [3] and extracted using Praat software [4] and the F0 contour information from the TANDEM-STRAIGHT object in Matlab [5]. F0 Glide = $F0_{End} - F0_{Start}$; Formant Dispersion (FormDisp) = ratio between consecutive formant means (from F1 to F4, maximum formant frequency set to 5.5 kHz, window length 0.025 s); HNR (harmonics-to-noise ratio) was extracted with the cross-correlation method (mean value; time step = 0.01 s; min pitch = 75 Hz; silence threshold = 0.1, periods per window = 1.0).

Table S19

Summary of the acoustic characteristics of male voice morphs separately for each Emotion and Morph Type

	MType	F0 Mean	F0 SD	F0 Glide	FormDisp	HNR
Happiness						
	Full	259	89	-74	999	17
	F0	259	89	-74	1037	17
	Timbre	158	21	-43	985	15
Pleasure						
	Full	121	18	-32	1064	14
	F0	121	18	-32	1046	15
	Timbre	158	21	-43	1058	14
Fear						
	Full	191	23	-19	1077	17
	F0	191	23	-19	1046	17
	Timbre	158	21	-43	1074	17
Sadness						
	Full	122	14	-47	1040	16
	F0	122	14	-47	1049	16
	Timbre	158	21	-43	1033	16
Average						
	Full	158	21	-43	1047	17

Note. All acoustical parameters were adapted from [3] and extracted using Praat software [4] and the F0 contour information from the TANDEM-STRAIGHT object in Matlab [5]. F0 Glide = $F0_{End} - F0_{Start}$; Formant Dispersion (FormDisp) = ratio between consecutive formant means (from F1 to F4, maximum formant frequency set to 5.5 kHz, window length 0.025 s); HNR (harmonics-to-noise ratio) was extracted with the cross-correlation method (mean value; time step = 0.01 s; min pitch = 75 Hz; silence threshold = 0.1, periods per window = 1.0).

3. Supplemental Design Information

Table S20

Summary of response key mappings to emotions

	**„d“**	**„f“**	**„j“**	**„k“**
CB 1	happiness	pleasure	sadness	fear
CB 2	sadness	fear	happiness	pleasure
CB 3	pleasure	happiness	fear	sadness
CB 4	fear	sadness	pleasure	happiness

Note. *Participants were instructed explicitly to press the keys „d“ and „f“ with their left index- and middle-finger and the keys „j“ and „k“ with their right index- and middle-finger. CB = counterbalancing condition.*

Table S21

Participant assignment to the different response key mapping

	**Singers**	**Instrumentalists**
CB 1	11	10
CB 2	10	12
CB 3	16	8
CB 4	8	13

Note. Participants were randomly assigned to key mappings. CB = counterbalancing condition.

References

1. van den Bergh, D., van Doorn, J., Marsman, M., Draws, T., van Kesteren, E.-J., Derks, K., Dablander, F., Gronau, Q.F., Kucharský, Š., and Gupta, A.R.K.N., et al. (2020). A Tutorial on Conducting and Interpreting a Bayesian ANOVA in JASP. L'Année psychologique *Vol. 120,* 73–96. 10.3917/anpsy1.201.0073.
2. Benjamini, Y., and Hochberg, Y. (1995). Controlling the False Discovery Rate: A Practical and Powerful Approach to Multiple Testing. Journal of the Royal Statistical Society: Series B (Methodological) *57,* 289–300. 10.1111/j.2517-6161.1995.tb02031.x.
3. McAleer, P., Todorov, A., and Belin, P. (2014). How do you say 'Hello'? Personality impressions from brief novel voices. PLoS One *9,* e90779. 10.1371/journal.pone.0090779.
4. Boersma, P. (2018). Praat: Doing phonetics by computer [Computer program]: Version 6.0.46, retrieved January 2020 from http://www.praat.org/. http://www. praat. org.
5. MATLAB (2020). version 9.8.0 (R2020a) (The MathWorks Inc).
